# Some Observations on the Pathology of the Spleen

**Published:** 1824-06

**Authors:** John Vetch

**Affiliations:** Member of the Medical Society of Edinburgh, and the Medico-Chirurgical and Geological Societies, London; Physician to the Charter-house, and late Physician to the Army.


THE LONDON
### Medical and Physical Journal.

6 OF VOL. LI.] JUNE, 1824. [NO. 304.

For many fortunate discoveries in medicine, and for the detection of numerous errors, the world is indebted to the rapid circulation of Monthly Journals; and there never existed any work, to which the Faculty, in Europe and America, were under deeper obligations, than to the Medical and Physical Journal of London, now forming a long, but an invaluable, series.—RUSH.

### ORIGINAL COMMUNICATIONS,
### SELECT OBSERVATIONS, &c.

ART. I.—*Some Observations on the Pathology of the Spleen.* By JOHN VETCH, M.D. F.R.S. E.; Member of the Medical Society of Edinburgh, and the Medico-Chirurgical and Geological Societies, London; Physician to the Charter-house, and late Physician to the Army.

THE unsuccessful attempts which have been made by physiologists to assign a direct or specific function to the spleen, and the difficulties which present themselves in making it the subject of experiment, hold out little prospect of our becoming, by that mode of investigation, better acquainted with the precise and immediate use of this viscus. As the alterations of structure which take place from disease are often more successful in explaining and extending our knowledge of minute anatomy, than the most expert and assiduous use of the knife, so, by an attentive study of pathology, we may often derive a degree and extent of information, more perfect than direct experiment can effect.

In all the operations of the material world, in which a chain of consecutive actions is established, we find that every such system has, within certain limits, a power of self-adjustment, proportioned to the vicissitudes of impulse to which it is subject, from the varying conditions of its external relations.

The various organs of the animal system are not only competent to their specific operations, but capable, by a reciprocity of function, of acting as compensating powers, when the operation of one or more is either increased or diminished. The power of assimilation and separation thus mutually influence each other, in their varying states of increased or diminished activity, preserving the existence of the animal under circumstances which would otherwise be incompatible with life. The suppression of one excretory function being counterbalanced by the increase of another, the system is thus enabled to maintain its integrity, under a great diversity of circumstances. There occur in the animal economy great and sudden changes in the distribution of the blood, such as, on this principle, would require a structure capable of guarding against effects, which, but for the familiarity with which we are accustomed to observe them, would seem incompatible with life, and which no organ, having a vital function to perform, could sustain with the same impunity as may be done by one destined to a secondary operation.

The spleen, independent of other uses, by its structure and situation, appears to be particularly fitted to afford that accommodation which the system requires, under the more sudden and violent changes which take place in the distribution of the blood; and, however important this viscus may be to the development of those changes which fit the blood to the purpose of assimilation, the consideration of its pathology, I think, will show that it also affords the means of protecting the system, under circumstances which suddenly destroy the equilibrium of the circulation; and, when repeatedly acted upon in this way, it becomes itself the source of other symptoms, the true character and nature of which have been too much disregarded.

We cannot expect to derive any certain information relative to the function of the spleen, under the disturbance which all direct experiments must necessarily create; and with its minute anatomy we still possess a very limited acquaintance. Its situation admits of easy distention, while its external wrinkled appearance indicates its capacity to undergo this, and is analogous to other structures fitted for such a purpose. Its artery is one of the first given off from the aorta, which is not exposed to the effects of external temperature. So long ago as the seventeenth century, the cellular structure of the spleen was satisfactorily demonstrated by Malphigi, with this peculiarity, however, which is easily ascertained,—that, while it readily admits of being distended by injecting the vein, the same result is not obtained by injecting the artery. Any power, therefore, which the arterial vessels may possess of filling the cells, has hitherto escaped the observation of the anatomist; and whether the cells become distended by a specific operation of the arterial vessels, or by a retrograde or obstructed course of the blood through the vein, is a subject for a separate inquiry. The present observations are confined to such data as the phenomena of disease are capable of furnishing.

The most evident, and perhaps the most familiar, example of a great and sudden change in the distribution of the quantity of blood that can be offered, is that which attends a paroxysm of ague. If the glare of a spurious philosophy had not directed the attention of the founders of sects from observing the simple phenomena of fever, the various speculations which have successively divided or commanded the suffrages of the medical public, would never have been heard of. If we confine ourselves strictly to the phenomena of the case, there can be no pretence for assuming any gratuitous state of the system to explain them. They form a connected series of actions, necessarily resulting in the order they assume, and corresponding with the powers inherent at all times in the several organs of the body. The blood, during the cold stage, being thrown upon the large vessels, will accumulate where the least resistance is opposed; and in this way, for the reasons I have given, the spleen is likely to receive a greater share than any other part; and that it does suffer more than any other part in cases of simple ague, is proved by dissection.

The accumulation of blood in the vessels of the trunk, and especially in the portal system, will proceed, until so much of the exciting force is expended as to permit its return to its usual state of equilibrium; but this will necessarily take place at an increased ratio of action, proportionable to the extent to which the superficial vessels have been emptied: besides, the supply being in all such cases regulated by the demand, and the superficial vessels having acquired an accumulated power of action from having been in a quiescent state, the return of blood into these vessels consequently acts with preternatural force, and establishes the hot stage. When the patient is cut off during the cold stage, and also when the protracted continuance of the hot stage indicates that such a change has taken place as to prevent the termination of the fever, in consequence of a new source of irritation having arisen, dissection invariably shows the earliest alteration to have taken place in the spleen: and, indeed, without some such system of adjustment,—some means of mitigating these sudden impressions, such as the passive structure of the spleen seems to present, it is difficult to conceive how the phenomena of ague could take place.

The human system requires, in a more particular manner, a resource against sudden changes in the distribution of the blood. Man enjoys, by the greater development of his cerebral system,* a more extensive range of temperature for his existence than any other animal; but, without a corresponding vascular organisation, he could not avail himself of that power to its full extent. The higher state of his intellectual faculties affords him another source of enjoyment, of which no other animal partakes; but these very faculties operate, too often, as so many disturbing causes to the regularity of the circulation: the effect of sudden emotions of the mind, and of depressing passions, is perhaps the most frequent of all the sources of splenic congestion.

---

* Vide Brodie's Experiments on Animal Temperature.

Thus far the spleen seems calculated to receive a great part of the shock which is produced by a sudden alteration in the balance of the circulation. I am far, however, from conceiving that this is any thing more than a contingent attribute of that organ, although its peculiar office will suffer such an interruption with, perhaps, a greater degree of impunity to the system, than would arise from the suspension of any other capable of affording, by its size, the same extent of relief. This very circumstance illustrates, by its effects, the nature of those changes which the blood undergoes by its due circulation through the spleen, in rendering it fit for the purposes of assimilation; as is indicated by the known changes in the state of the urinary secretion, produced by the interruption occasioned by the cold stage of fever, and the restoration of its office towards the close of the paroxysm. In the first there is an absence of almost all its constituent principles; and, in the second, by a redundancy in proportion to their antecedent retention, and more especially by the great quantity of lithic acid and urea now contained in it. These relative changes we shall have again occasion to notice, as also produced by more chronic derangements of the spleen.

The spleen, as compared with the other viscera, is not prone to acute inflammation; and, where it does occur, it has its seat in the external coat; but, as this, like the viscus itself, admits of great distention, the inflammation is not accompanied with the same degree of pain which attends it in less distensible parts: neither is it so much under the command of depletion, and hence it is very liable to terminate fatally. The left kidney very frequently sympathises with every variety of splenic congestion. The principal purpose of these observations is, however, to call attention to certain chronic derangements of function and structure, which are every day mistaken for those of other parts.

Formerly, almost every impaired state of the digestive functions was attributed to a deficiency of tone, or power, of the stomach and duodenum; and if, under such an impression, our predecessors failed to relieve their patients, by adopting such a belief, and by arranging the symptoms under the convenient term of dyspepsia, they exonerated themselves from the trouble of much further deliberation. Although the natural state of these organs remains very much *in statu quo*, we cannot say as much for the stability of medical opinion. The present fashion, which refers almost all symptoms arising from imperfect digestion to a diseased or impaired condition of the liver, is one of those examples of the empirical state to which the practice of medicine is subject, even in this age and country. A doctrine or practice, when powerfully illustrated by an enlightened individual, no sooner obtains

2

the credit due to it, than its abuse leaves it doubtful whether it is to be hailed as a blessing, or as a new and prolific source of empirical practice. Purgatives, though perhaps the most important class of medicines in the treatment of almost all diseases, were for a time much disused ; and is it not possible that they are now too unsparingly administered ? Much as I value this class of medicines, I am persuaded they have been adopted too exclusively. I have found the greatest benefit from emetics, when purgatives have completely failed, and more especially in some late cases of hemicrania, with slight paralytic affections. In like manner, I am persuaded that patients labouring under various symptoms of imperfect assimilation, have had their complaints further aggravated by the habitual use of mercurial preparations.

So far from the symptoms of dyspepsia being such as always to warrant a trial of any one form of medical treatment, there are none which require more discrimination in order to obtain a knowledge of the true seat of the disease, and where the very similitude presents difficulties which are unknown to the unthinking. The effects of an enlargement or obstruction of the spleen are such as are most universally referred to the liver, and yet the distinction is obvious enough when the practitioner is prepared to examine the difference, as well as the similitude, which they respectively exhibit.

In enlargement of the spleen, the patient seldom or never complains of much pain in the situation where it might be expected ; his appetite is generally good, yet his powers of assimilation are obviously deficient ; he loses flesh, and is incapable of any muscular exertion ; his features have a peculiar dark, bilious, or mahogany hue, but the conjunctiva preserves its white and healthy appearance ; perspiration is in time wholly suspended, and the skin acquires the appearance and feel of satin ;* the lips are pale, and there is generally much wasting of the gums ; the urine is limpid, and secreted very rapidly, but contains little or no urea. The patient's mind is generally morose and desponding. All these symptoms of a deranged condition of the spleen prove that, although the patient may exist for a long time without those changes going on in the blood which would otherwise take place, the powers of life cease to be renewed. An attack of epistaxis,† or the appearance of moisture upon the skin, are generally signs of returning health. The symptoms I have mentioned are commonly attended with coldness of the lower extremities, generally towards evening, and the pulse is quicker than natural.

Before discussing the treatment to be pursued, I shall revert

* This is observable in convalescents from the remittent fever of warm climates. Vide Jackson *on Febrile Diseases.*

† This was very common with convalescents from the Walcheren fever.

to the more generally exciting causes. A state of the system, such as I have now rapidly described, is often found in connexion with amenorrhœa; and, in such cases, an occasional acute pain is liable to occur in the region of the spleen, on any slight exertion. A determination of blood to the spleen is often vicarious with discharges from other parts, and, in cases where this has long obtained, the viscus has often acquired a very prodigious enlargement of its size.*

The enlargement of the spleen, in cases of long-continued ague, and after remittent fever of warm climates, is so well established by post-mortem examination, as to render particular reference to past experience unnecessary. In the only three cases in which I have seen a fatal termination during the cold stage of fever, the spleen was so much distended, and its structure so much impaired, that it appeared like a mass of dark, thick, and uncoagulated blood, which crumbled on the application of the finger; and the symptoms which so often supervene upon remittent fever, bear a much greater resemblance to those I have described, than any which have a direct reference to a diseased condition of the liver. Whenever a determination of the blood takes place, with undue force, to the portal system, the spleen seems to sustain the principal part of the shock, without permanent injury; but, by frequent repetition, it becomes subject to distention beyond its power of recovery. This I have endeavoured to illustrate by a reference to the effect of the exciting cause of ague; there is also a particular form of rheumatism, which is nothing less than a modification of the first-mentioned disease, and is equally the source of splenic distention. The complaint to which I allude has been endemic in many places, from which ague has disappeared in consequence of drainage and cultivation. It is simply a rheumatic affection of the muscular fibres, and is accompanied by a great want of energy in the cutaneous circulation. It may be said to have a cold and hot stage, inasmuch as the pain is regularly preceded by coldness of those parts and of the lower extremities, and proves a most tedious and untractable form of disease, and almost invariably serves to illustrate the effects of an irregular distribution of the blood in the production of splenic enlargements.

The most frequent, however, of all exciting causes may be fairly ascribed to mental depression and anxiety; and, arising from these, its symptoms are, perhaps, more simulative of other diseases than when induced by external agents. In very many cases in this metropolis, how often have patients gradually sunk under the operation of mercury, given for no other reason than that, as the practitioner could not assign a sufficient cause for

---

* Vide Morgagni.

the symptoms, he has placed the onus on the liver. The temporary relief which is procured by such doses of mercury as operate upon the bowels, have led a vast number of valetudinarians into the pernicious custom of having recourse to calomel or blue-pill on every aggravation of their symptoms, until they finally sink under accumulating infirmity. In all cases where the spleen is much implicated, a very limited experience will convince any man, who is willing to be taught, that any continued course of mercury, so far from proving of ultimate benefit, renders the patient less capable of regaining that general tone of the system, the want of which forms the principal obstacle to his recovery.

Enlargement of the spleen is often accompanied by local symptoms of such urgency, as to render evacuations indispensable; but a great part of the treatment must consist in obtaining an equable state of the circulation, and at the same time guarding against all causes of excitement, until we have removed every symptom of local congestion: to give tone to the stomach, and at the same time to maintain a solvent state of the intestines, are the primary objects to be attempted. Medicine, unassisted by strict dietetic system, will be of little avail; but, combined with attention to diet, sleep, exercise, friction, and bathing (at a temperature decreasing as the patient recovers his powers), often restore health from the lowest state of debility. Under exacerbations or return of the more troublesome symptoms, local blood-letting and alvine evacuations will be necessary. Flatulent food, including all unblanched vegetables, imperfectly fermented liquors, and all direct stimuli, must be avoided, especially until the skin has regained its functions. It is equally necessary that the patient, whose appetite is generally good, though he derives no nourishment from its gratification, should avoid the full distention of his stomach.

It is, perhaps, difficult to find any one article of the materia medica, which possesses any great tonic power, which will not in a short time require to be discontinued, by its diminishing either alvine discharge or the secretion of urine. I have, however, been fortunate in finding one very applicable to such a state of disease as I have endeavoured to describe: it is valuable as a tonic, and not less so from the diuretic power which it never fails to exert, if given in the form to which I confine its use. The article on which I have bestowed this panegyric, is a weak infusion of the leaves of the arbutus uva ursi: if the powder of the leaves is substituted, it produces a bitter much too intense to answer the end proposed. I was led to use this medicine extensively, both with officers and soldiers, who had laboured under protracted disease after the expedition to Walcheren. My subsequent experience has given me ample opportunities of appreciating its value as a medicine, in all cases resembling those I have mentioned. The successful application of small blisters to the epigastric region and left hypochondrium, and the insertion of a seton, are highly useful auxiliaries.

I have now said enough to induce some to take a more attentive view of the symptoms attending cases where the system seems to have lost its due power of assimilation. Hysteria, hypochondriasis, mental aberrations, and *diabetes*, are to be found arising out of primary obstruction of the function of the spleen; as, without the co-operation of this viscus, the blood ceases to acquire its recrementitious properties, none of the secretions possess their natural constituent parts; and, what is also worthy of notice, external injuries do not produce the secretion of what is termed healthy pus. The spleen, therefore, serves this one important function, which Mæbius has thus expressed: "sanguinem fæculentiorem ulterius elaborans et ita disponens, ut partes recrementosæ, salinæ et serosæ in locis convenientibus seperari queant." An obstruction to an office so essential to the formation of perfect blood, when it does not give rise to one of the secondary diseases which have been mentioned, terminates in what may be considered pure cachexy from defective assimilation. These observations cannot be better concluded than by the following quotation from Celsus, as an ample confirmation of the treatment I have recommended: "Dulcia omnia inimica sunt: item lac et caseus. Acida autem maxime conveniunt. Potest etiam dari vinum tenue austerum," (old hock or moselle in moderation,) "omniaque in cibis et potionibus, *quæ urinæ movendæ sunt.*" (Vide *de Lienosis*, cap. ix.)

